# Association between dietary vitamin A intake from different sources and non-alcoholic fatty liver disease among adults

**DOI:** 10.1038/s41598-024-52077-5

**Published:** 2024-01-22

**Authors:** Can Liu, Xiaona Sun, Jing Peng, Haiqing Yu, Jiao Lu, Yihui Feng

**Affiliations:** 1 School of Public Health, Shanxi Medical University, Taiyuan, China; 2 School of Management, Shanxi Medical University, Taiyuan, China; 3 Department of Respiratory and Critical Care Medicine, Qingdao Central Hospital, University of Health and Rehabilitation Sciences, Qingdao, China; 4 Department of Pediatrics, Qingdao Central Hospital, University of Health and Rehabilitation Sciences, Qingdao, China; 5 Shenzhen Maternity and Child Healthcare Hospital, Shenzhen, China; 6 School of Public Policy and Administration, Xi’an Jiaotong University, Xi’an, China; 7 School of Rehabilitation Science and Engineering, University of Health and Rehabilitation Sciences, Qingdao, China

**Keywords:** Diseases, Health care, Medical research, Risk factors

## Abstract

Non-alcoholic fatty liver disease (NAFLD) has become an urgent public health issue with high global prevalence, but data on NAFLD are inconsistent. The association of total dietary vitamin A intake with the NAFLD risk was not well documented in previous studies. To explore the relationship between dietary vitamin A intake from different sources and NAFLD risk among American adults. Data were collected from the National Health and Nutrition Examination Survey (NHANES) from 2007 to 2014. Logistic regression and restricted cubic spline models were used to estimate the relationship between total dietary vitamin A intake and NAFLD risk. 6,613 adult participants were included. After adjusting potential confounders, the odds ratios (ORs) with 95% confidence intervals (CIs) of NAFLD for the highest quartile intake of total vitamin A, preformed vitamin A, provitamin A carotenoids were respectively 0.86 (0.69–1.06), 0.97 (0.74–1.28), and 0.78 (0.61–0.99), compared to the lowest quartile. Stratifying gender and age, provitamin A carotenoids intake was inversely associated with NAFLD risk in females and participants aged < 45 years. Dose–response analysis indicated a linear negative relationship between provitamin A carotenoids intake and NAFLD risk. Provitamin A carotenoids intake was inversely associated with NAFLD, especially in women and those aged < 45 years among adult American.

## Introduction

Non-alcoholic fatty liver disease (NAFLD) is characterized by lipid accumulation in the liver without excessive alcohol intake or any other liver diseases^1^. NAFLD encompasses a wide spectrum of liver damage, ranging from simple liver steatosis to non-alcoholic steatohepatitis (NASH), liver fibrosis, cirrhosis and hepatocellular carcinoma (HCC)^2–5^, which increases the risk of hypertension, diabetes, obesity, and cardiovascular diseases^6–8^. The prevalence of NAFLD is estimated to be approximately 30% among American adults^9^. To our knowledge, there is currently no pharmacological treatment approved for NAFLD^10^. Dietary modifications and antioxidants have been recommended to prevent the progression of NAFLD^11^.

Several modifiable lifestyles and dietary factors are associated with chronic diseases. Previous studies demonstrated that high intake of vegetables, fruits, and whole grains, as a dietary pattern was associated with reduced risk of hypertension, hyperuricemia, type 2 diabetes, and cardiovascular diseases^12–16^. In addition, several studies reported that consuming fried foods, refined grains, processed meat, and fructose-rich foods increased the risk of NAFLD^17–19^. In contrast, whole grains, legumes, probiotic dairy products, vegetables, and fruits reduced the risk of NAFLD^19–22^.

Vitamin A is a common dietary antioxidant^23^ with both antioxidant and antifibrotic properties^24^. The two major forms of dietary vitamin A are preformed vitamin A (such as retinol and retinyl esters) and provitamin A carotenoids (such as β-carotene). Animal products with preformed vitamin A provide ≥ 70% of daily vitamin A intake. Provitamin A carotenoids that are mainly found in fruits and vegetables provide ≤ 30% of daily vitamin A intake^25^, which can be cleaved and metabolized into retinol after absorption by the intestinal cells^26^. The basic mechanisms of preformed vitamin A and carotenoids absorption were first investigated 40 years ago using rat everted intestinal sacs^27–29^. The data obtained indicated that preformed vitamin A absorption occurred via (a) carrier-dependent proteins, while carotenoids were absorbed by a passive diffusion process ^26^. In addition, preformed vitamin A is absorbed by intestinal epithelial cells, stored in the liver, and metabolized into retinoic acid (RA) and retinyl esters (RE) in target cells^30^. In liver cells, RE are hydrolyzed by retinyl ester hydrolase to produce retinol, and RA can reduce the progression of NAFLD by increasing triglyceride hydrolysis and fat oxidation^30^. Carotenoids possess antioxidant properties and can physiologically scavenge free radical species in the liver, thereby ameliorating hepatic dysfunction^31^. Therefore, it is important to study the effects of dietary vitamin A on NAFLD patients. At present, the potential relationship between total dietary vitamin A intake and NAFLD risk remains elusive. An Iranian study indicated that higher vitamin A intake was associated with reduced risk of NAFLD^32^. Based on the results of the study of Vahid et al.^33^, dietary vitamin A was associated with a decreased the risk of NAFLD. Additionally, a Chinese cohort revealed an inverse relationship between vitamin A intake and NAFLD risk^34^. However, a Korean study showed that participants with NAFLD had higher vitamin A intake than healthy subjects^35^. Similarly, Federico et al. reported higher vitamin A intake in NASH subjects than those without NASH^36^.

Previous studies mainly focused on the association between total dietary vitamin A intake and NAFLD risk^32–34^. However, studies reporting the association between dietary vitamin A intake from different sources and NAFLD risk are scarce. Furthermore, the dose–response relationship between dietary vitamin A intake and NAFLD risk has not been previously investigated. The present cross-sectional study aimed to explore the relationship between dietary vitamin A intake from different sources and NAFLD risk. The results of our study may be useful for alleviating NAFLD in American adults.

## Methods

### Study population

The National Health and Nutrition Examination Survey (NHANES) was conducted by the National Center for Health Statistics (NCHS) of the Centers for Disease Control and Prevention (CDC) in the United States (US)^37^. The survey was a continuous program with 2-year cycles starting from 1999^38^. The NHANES included demographic, socioeconomic, dietary, and health-related questions. The examinations consisted of medical, dental, and physiological measurements, as well as laboratory tests conducted by highly trained medical personnel, which were collected from a complex multistage stratified sample representative of the non-institutionalized civilian US population^39^. The study protocol was approved by the Research Ethics Review Board of National Center for Health Statistics. In addition to this, we confirmed that all research was performed in accordance with relevant guidelines/regulations, and included in our manuscript a statement confirming that informed consent was obtained from all participants and/or their legal guardians.

**Figure 1:**
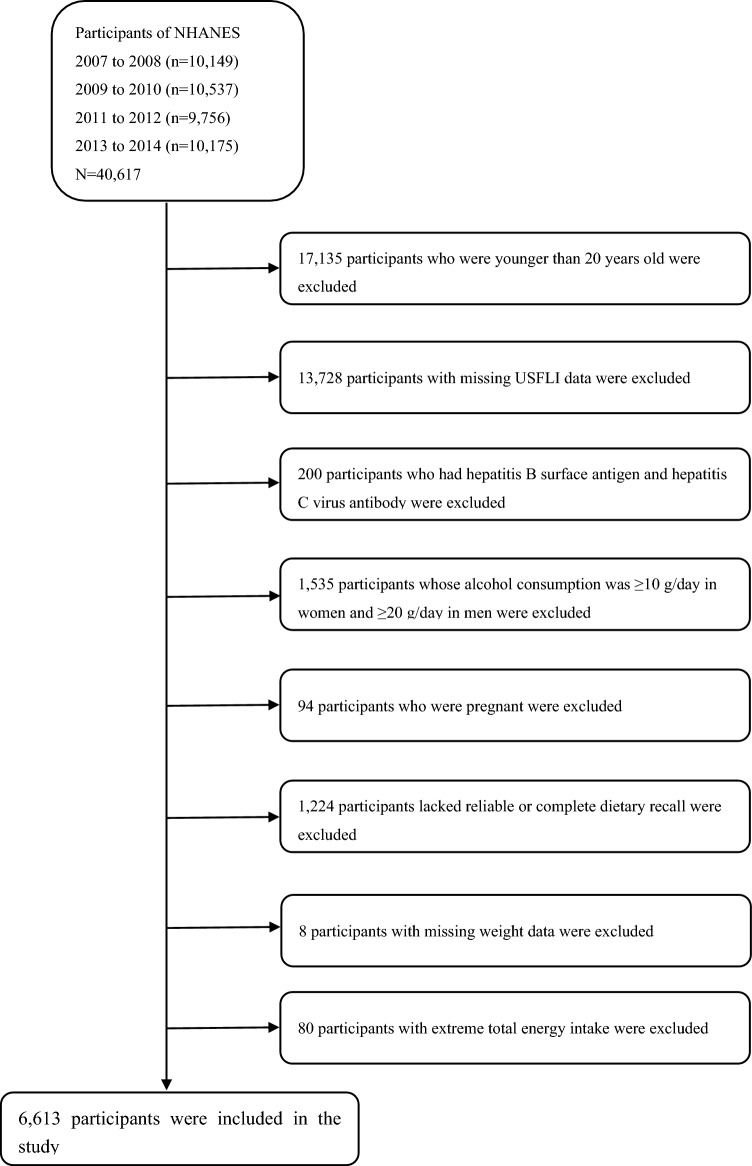
Flow chart showing the eligible participants selection of the study. *NHANES* National Health and Nutrition Examination Survey.

This study analyzed the continuous NHANES data between 2007 and 2014. The NHANES database included 40,617 participants (20,180 males and 20,437 females), and 23,482 subjects aged 20 years or older were included. Participants with missing information to calculate the United States fatty liver index (USFLI) were excluded (n = 13,728). Furthermore, 200 individuals who had hepatitis B surface antigen and hepatitis C virus antibodies were excluded. Subsequently, participants whose alcohol consumption was ≥ 10 g/day in women and ≥ 20 g/day in men (n = 1535) were also excluded. Finally, 6613 participants (3067 males and 3546 females) were included in our analyses, after excluding those who were pregnant (n = 94), lacking reliable or complete dietary recall (n = 1224), missing weight data (n = 8) and whose average energy intake was higher than mean + 3 standard deviations (SDs) or less than mean – 3 SDs (n = 80) (Fig. 1).

### NAFLD definition and measurement

According to a previous study^40^, NAFLD was defined based on USFLI, which was previously validated and excluded those with other causes of chronic liver disease and exposure to steatogenic medication. USFLI was calculated based on age, race, waist circumference, fasting glucose level, gamma glutamyl transferase level, and fasting insulin level^41^. A USFLI cut-off value of ≥ 30 indicated the presence of NAFLD^41^. As reported in previous articles, USFLI is a credible non-invasive measure of NAFLD and an independent predictor of liver-related and overall mortality^42–44^. The USFLI was calculated as follows:

$$\begin{aligned} USFLI & = (e \hat (-0.8073 * non{-}Hispanic\, Black + 0.3485 * Mexican\, American + 0.0093 * Age + 0.6151 * ln (GGT) + 0.0249 * Waist \, Circumference +1.1792 * ln (Insulin) + 0.8242 * ln (Glucose) - 14.7812)/\\ &\quad((1 + (e \hat (-0.8073 * non{-}Hispanic\, Black + 0.3485 * Mexican \, American + 0.0093 * Age + 0.6151 * ln (GGT) + 0.0249 * Waist \, Circumference +1.1792 * ln (Insulin) + 0.8242 * ln (Glucose) - 14.7812))) * 100 \end{aligned}$$

The value for “non-Hispanic Black” and “Mexican American” is 1 if the person is of that ethnicity, and 0 if the person is not.

### Dietary vitamin A intake

Dietary vitamin A intake was calculated by two 24-h dietary recall interviews in retinol activity equivalents (μg)^45^. The first dietary recall interview was performed in person at the mobile examination center, and the second interview was performed through telephone after 3–10 days. Nutrient intake was calculated according to the US Department of Agriculture’s Dietary Research Food and Nutrition Database for Dietary Studies^15^. Different sources of dietary vitamin A, such as preformed vitamin A (milk and milk products, meat, poultry, fish, eggs) and provitamin A carotenoids (legumes, nuts and seeds, grain products, fruits, vegetables) were identified using the food codes^46^. The dietary vitamin A intakes from the two 24-h recalls were averaged and adjusted to the energy intake for subsequent analysis. Dietary vitamin A intake (μg/1000 kcal/day) was divided into quartiles. It should be noted that dietary vitamin A intake from supplements was not included in total dietary vitamin A intake. As for preformed vitamin A, 1 μg retinol activity equivalents (RAEs) were equal to 1 μg all-trans retinol from animal foods. Provitamin A carotenoids was estimated by using the diet equation: 1 RAEs (μg) = 1/12 β-carotene (μg) + 1/24 other provitamin A (μg)^45^.

### Covariates

Potential confounding factors were adjusted in multivariate models, including gender (male and female), age (20–44 years, 45–59 years, 60–74 years and ≥ 75 years), race (Mexican-Americans, other Hispanics, non-Hispanic Whites, non-Hispanic Blacks and other races), body mass index (BMI) (normal: < 25 kg/m^2^, overweight: 25 to < 30 kg/m^2^,obese: ≥ 30 kg/m^2^), education level (under high school, high school, and above high school), annual household income (< $20,000, $20,000–$44,999, $45,000–$74,999 and ≥ $75,000), smoking status (smoking at least 100 cigarettes in life or not), vigorous recreational activity (yes or no), diabetes (yes or no), hypertension (yes or no), total cholesterol (TC), and uric acid (UA). Diabetes was defined based on the following conditions: (1) fasting blood glucose level ≥ 7.0 mmol/L, (2) 2-h plasma glucose level ≥ 11.1 mmol/L, (3) use of antidiabetic pills or insulin, or (4) self-reported diabetes diagnosed by a physician^47^. Hypertension was defined as mean systolic blood pressure ≥ 130 mmHg or mean diastolic blood pressure ≥ 80 mmHg, and taking antihypertensive agents and self-reported physician diagnosis^48^.

### Statistical analysis

All statistical analyses were performed using Stata 15.0. Appropriate sample weights and units and stratified information of the sample design were used in our analyses. In accordance with the NHANES guidelines^49^, new 8-year sample weights were computed by dividing the 2-year dietary weights by four. To compare the difference between groups, Chi-square test was used for categorical variables, while Student’s t-test or Mann–Whitney U test was used for continuous variables normal and non-normal distribution. Logistic regression models were used to evaluate the association between dietary retinol and NAFLD risk. For these analyses, the dietary vitamin A levels were divided into quartiles, and the first quartile (Q1) was set as a reference category. Two models were constructed: model 1 included gender and age; in model 2, we further adjusted for race, educational level, smoking status, recreational activities, annual household income, hypertension, diabetes, BMI, UA, and TC levels. After stratification by sex (male and female) and age (20–44 and ≥ 45 years), logistic regression analyses were conducted to analyze the relationship between total dietary vitamin A intake and NAFLD risk. Odds ratios (ORs) with 95% confidence intervals (CIs) were computed based on the results of logistic regression analyses. Furthermore, dose–response relationships between total dietary vitamin A intake and NAFLD risk were assessed using a restricted cubic spline regression and three knots were located at the 5th, 50th, and 95th percentiles. The non-linearity *p*-value was computed by examining the values of the quadratic zero spline coefficient, and a two-tailed *p* < 0.05 was considered statistically significant.

### Ethics statement

The participants provided written informed consent for publishing any potentially identifiable images or data in this study.

## Results

The mean age of participants was 50.75 years. The mean level of dietary vitamin A was 334 μg/1000 kcal/day (305 and 359 μg/1000 kcal/day in males and females, respectively). NAFLD was detected in 36.7% of participants (41.9% of males and 32.1% of females). Table 1 showed the comparison of baseline characteristics between the NAFLD and non-NAFLD groups (stratification by gender shown in Supplementary Table S1). Participants with NAFLD tended to be older, involved more Mexican-Americans, and had lower education level, annual household income, vigorous recreational activity, and provitamin A carotenoids intake compared to the control group. In addition, participants with NAFLD were more likely obese, smokers, hypertensive, diabetic, and hyperuricemic and had higher preformed vitamin A intake than those without NAFLD (all *p* < 0.01).

**Table 1:** Baseline characteristics of the participants by NAFLD, U.S. adults aged ≥ 20 years, NHANES 2007–2014.

Group	NAFLD (total)		p-value
	No	Yes	
Age group (n, %)			< 0.001
20–44 years	1890(45.12%)	709(29.25%)	
45–59 years	988(23.59%)	655(27.02%)	
60–74 years	862(20.58%)	742(30.61%)	
≥ 75 years	449(10.72%)	318(13.12%)	
Race/ethnicity (n, %)			< 0.001
Mexican American	488(11.65%)	552(22.77%)	
Other Hispanic	444(10.60%)	294(12.13%)	
Non-Hispanic White	1845(44.04%)	1155(47.65%)	
Non-Hispanic Black	931(22.22%)	269(11.10%)	
Other/multiracial	481(11.48%)	154(6.35%)	
BMI (n, %)			< 0.001
< 25 kg/m^2^	1716(41.02%)	108(4.46%)	
25 to < 30 kg/m^2^	1568(37.49%)	637(26.32%)	
≥ 30 kg/m^2^	899(21.49%)	1675(69.21%)	
Educational level (n, %)			< 0.001
< High school	902(21.55%)	802(33.14%)	
High school	954(22.80%)	543(22.44%)	
> High school	2329(55.65%)	1075(44.42%)	
Annual household income (n, %)			< 0.001
< $20,000	780(19.45%)	561(24.14%)	
$20,000–$44,999	1352(33.71%)	931(40.06%)	
$45,000–$74,999	781(19.47%)	409(17.60%)	
≥ $75,000	1098(27.37%)	423(18.20%)	
Smoking status (n, %)			< 0.001
Yes	1576(37.64%)	1131(46.66%)	
No	2611(62.36%)	1293(53.34%)	
Vigorous recreational activity (n, %)			< 0.001
Yes	1031(24.61%)	270(11.14%)	
No	3158(75.39%)	2154(88.86%)	
Hypertension (n, %)			< 0.001
Yes	1648(39.34%)	1539(63.49%)	
No	2541(60.66%)	885(36.51%)	
Diabetes (n, %)			< 0.001
Yes	505(12.06%)	910(37.54%)	
No	3684(87.94%)	1514(62.46%)	
Cholesterol (mg/dL)	192.16 ± 41.19	194.23 ± 41.79	0.051
Uric acid (mg/dL)	5.14 ± 1.30	6.03 ± 1.43	< 0.001
Average energy intake (kcal/day)	1903.27 ± 695.88	1912.03 ± 711.51	0.625
Total dietary vitamin A intake (RAEs, μg/1000 kcal/day)	338.66 ± 284.09	325.69 ± 279.77	0.072
Preformed vitamin A intake (RAEs, μg/1000 kcal/day)	122.70 ± 150.62	134.97 ± 205.69	0.005
Provitamin A carotenoids intake (RAEs, μg/1000 kcal/day)	198.80 ± 238.80	171.24 ± 181.11	< 0.001

The associations of total dietary vitamin A, preformed vitamin A, and provitamin A carotenoids intake with NAFLD risk were shown in Table 2. In the univariate logistic regression model, the highest quartile of total dietary vitamin A intake (OR: 0.79, 95% CI: 0.67–0.92) and provitamin A carotenoids intake (OR: 0.64, 95% CI: 0.52–0.79) were inversely associated with NAFLD risk compared to the lowest quartile of intake. However, there was no significant association between preformed vitamin A intake and NAFLD risk. After adjusting age and gender, the association remained significant: 0.70 (95% CI: 0.58–0.84) and 0.59 (95% CI: 0.47–0.74) for total dietary vitamin A and provitamin A carotenoids intake, respectively. Furthermore, after adjusting race, educational level, smoking status, recreational activities, annual household income, hypertension, diabetes, BMI, UA and TC levels, the association remained significant for provitamin A carotenoids intake: 0.78 (95% CI: 0.61–0.99, *p* < 0.05). However, the protective effect of total dietary vitamin A intake on NAFLD was no longer significant after adjusting the above covariates (model 2).

**Table 2:** Weighted ORs and 95% CIs for NAFLD according to the quartiles of dietary retinol intake (μg /1000 kcal/day).

	Crude, OR (95% CI)	p-trend	Model 1, OR (95% CI)	p-trend	Model 2, OR (95%CI)	p-trend
Total dietary vitamin A intake (RAEs, μg/1000 kcal/day)		0.004		< 0.001		0.103
< 191.63	1.00(ref.)		1.00(ref.)		1.00(ref.)	
191.63 to < 284.53	1.11(0.92–1.34)		1.03(0.84–1.26)		1.08(0.80–1.44)	
284.53 to < 418.94	1.06(0.91–1.23)		0.96(0.81–1.15)		1.01(0.79–1.29)	
≥ 418.94	0.79(0.67–0.92)**		0.70(0.58–0.84)**		0.86(0.69–1.06)	
Preformed vitamin A intake (RAEs, μg/1000 kcal/day)		0.217		0.773		0.655
< 59.27	1.00(ref.)		1.00(ref.)		1.00(ref.)	
59.27 to < 104.86	1.10(0.92–1.31)		1.02(0.86–1.22)		1.12(0.88–1.42)	
104.86 to < 164.21	1.21(1.02–1.44)*		1.13(0.95–1.34)		1.12(0.88–1.43)	
≥ 164.21	1.11(0.92–1.32)		1.02(0.83–1.24)		0.97(0.74–1.28)	
Provitamin A carotenoids intake (RAEs, μg/1000 kcal/day)		< 0.001		< 0.001		0.011
< 70.37	1.00(ref.)		1.00(ref.)		1.00(ref.)	
70.37 to < 138.53	0.95(0.78–1.15)		0.94(0.77–1.16)		1.08(0.84–1.38)	
138.53 to < 253.07	0.85(0.70–1.04)		0.82(0.67–1.01)		0.93(0.70–1.23)	
≥ 253.07	0.64(0.52–0.79)**		0.59(0.47–0.74)**		0.78(0.61–0.99)*	

*OR* odds ratio, *CI* confidence interval.

Model 1 adjusted for gender and age. Model 2 adjusted gender, age, race, education level, smoking status, physical activity, income level, hypertension, diabetes, BMI, UA and TC. The lowest quartile of dietary retinol intake was used as the reference group. Results are survey-weighted. *p < 0.05, **p < 0.01. Test for trend based on variable containing median value for each quartile.

Supplementary Tables S2 and S3 show the association between dietary vitamin A intake and NAFLD risk in the subgroup analyses stratified by gender and age, respectively. In multivariate-adjusted model 2, the highest quartile of provitamin A carotenoids intake (OR: 0.61, 95% CI: 0.43–0.89) was negatively associated with NAFLD risk only in females. In Supplementary Table S3 (model 2), provitamin A carotenoids intake had 40% lower odds of NAFLD risk (OR: 0.60, 95% CI: 0.42–0.87, the highest vs. lowest quartile of provitamin A carotenoids intake) among participants aged < 45 years. After adjusting potential confounding factors in model 2, there were no significant associations of total dietary vitamin A, preformed vitamin A, and provitamin A carotenoids intake with NAFLD risk among participants ≥ 45 years of age.

**Figure 2:**
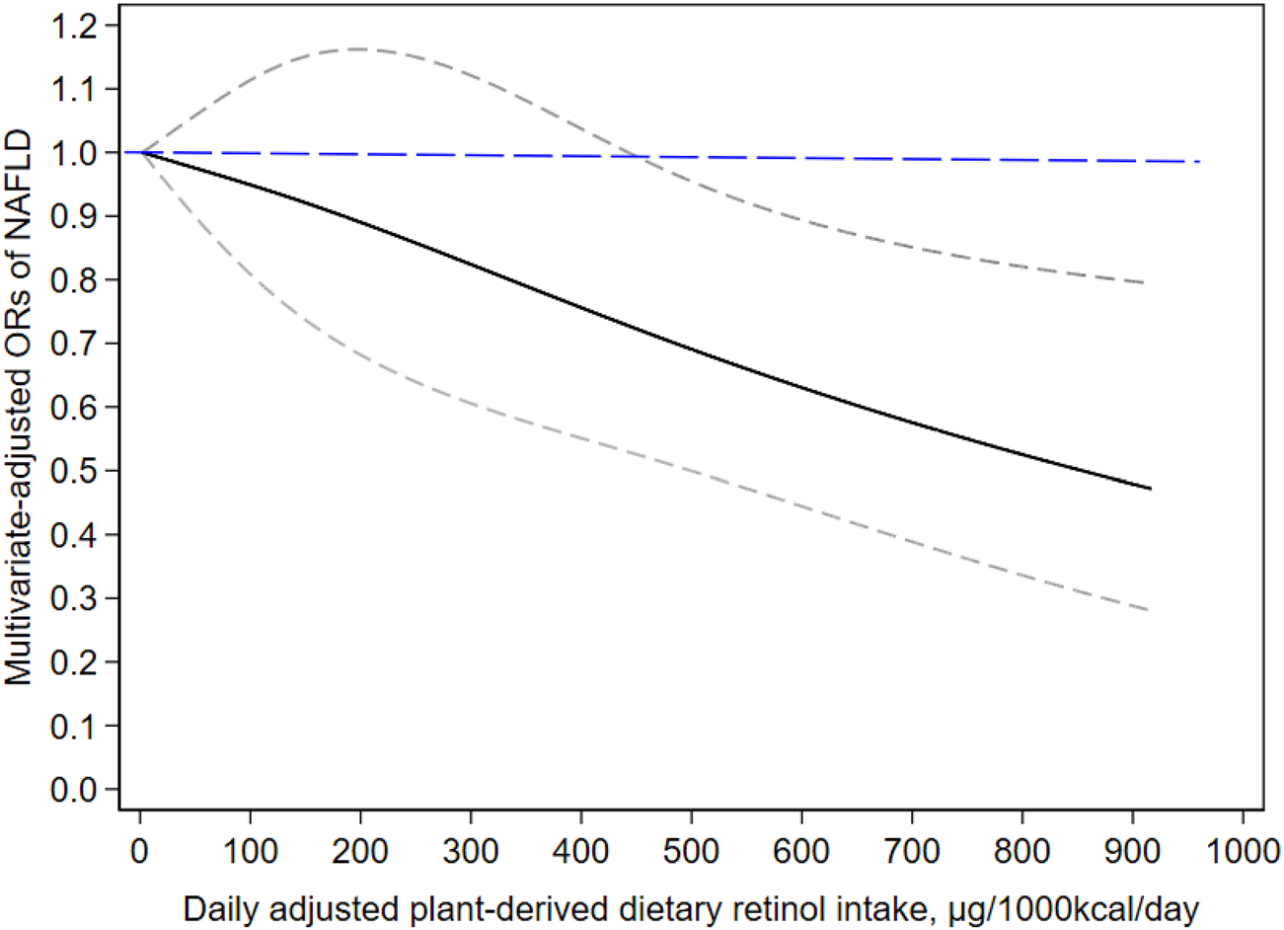
The dose–response pattern of plant-derived dietary retinol intake exposures and the NAFLD status was displayed in the restricted cubic splines model. The results were presented treating the lowest level of plant-derived dietary retinol intake (2 μg/1000 kcal/day) as a reference group. Potential confounding factors, such as gender, age, race, education level, smoking status, physical activity, income level, hypertension, diabetes, BMI, UA and TC were adjusted. The solid line and dashed line represent the estimated odds ratio (ORs) and the corresponding 95% confidence intervals (CIs), respectively.

The dose–response relationship between provitamin A carotenoids intake and NAFLD risk is displayed in Fig. 2. A linear negative dose–response correlation was observed between provitamin A carotenoids intake and NAFLD risk (P _for non-linearity_ = 0.734). When provitamin A carotenoids intake reached 449 μg/1000 kcal/day, it was inversely associated with NAFLD (OR: 0.72, 95% CI: 0.53–0.99).

## Discussion

Total dietary vitamin A intake was not associated with NAFLD risk after adjusting potential confounders. However, there was a significant association between the dietary vitamin A intake from different sources and NAFLD risk, in which provitamin A carotenoids intake was inversely associated with the risk of NAFLD. After stratification by gender and age, the significant correlations remained in both females and participants aged less than 45 years. A linear negative dose–response relationship was also found between provitamin A carotenoids intake and NAFLD risk. To the best of our knowledge, this is the first study to demonstrate the relationship between dietary vitamin A intake from different sources and NAFLD risk.

Several studies reported contradictory results about the association between dietary vitamin A intake and NAFLD risk. A population-based study in Iran indicated that the consumption of dietary vitamin A could decrease the risk of NAFLD^32^. Vahid et al.^33^ reported that dietary vitamin A intake was negatively related to the risk of NAFLD. Moreover, a cohort study involving 241 Chinese rural adults also found a negative association between dietary vitamin A intake and NAFLD risk^34^. Conversely, a Korean study with 80 participants showed that the dietary intake of vitamin A was higher in NAFLD patients than in healthy subjects^35^. Another study in Italy also reported similar results^36^. These controversial findings might be attributed to the lack of adjustment for potential confounders as well as discrepancies in ethnic background, dietary patterns, and study design.

Provitamin A carotenoids are widely distributed in plant foods. In this study, provitamin A carotenoids (such as β-carotene) was assessed by calculating vitamin A mainly from plant foods such as legumes, beans, fruits, and vegetables. Previously, few studies had reported the association between dietary vitamin A intake from different sources with NAFLD risk. Our results found that provitamin A carotenoids intake was inversely associated with NAFLD. Although the mechanism underlying the association between provitamin A carotenoids intake and NAFLD risk remains unclear, some possible mechanisms have been proposed. Higher mobilization of β-carotene for conversion into retinol could be responsible for the lower intake of provitamin A carotenoids in NAFLD patients than in healthy subjects in the present study^50^. Another possible explanation might be that the bioavailability of carotenoids was greatly affected by food substrate^51–54^. Experimental studies showed that carotenoids could reverse steatosis, inflammation, and fibrosis progression in NASH, thereby preventing macrophage or Kupffer cell activation, attenuating insulin resistance, and ameliorating steatohepatitis^55^. Through their anti-inflammatory and antioxidant properties, carotenoids modulate intracellular signaling pathways involved in gene expression and protein translation^56^. A previous study indicated that the antioxidant properties of carotenoids could prevent liver damage and NAFLD by alleviating oxidative stress in hepatocytes^57^. Several studies reported that the levels of high-sensitivity C-reactive protein (hs-CRP) and inflammatory cytokines (e.g., IL-6 and TNF-α) were associated with NAFLD risk, which were considered as biomarkers of inflammation leading to endothelial cell damage^58,59^. Several studies demonstrated that carotenoids and their metabolites were likely to modulate adiponectin expression^60,61^. Adiponectin may alleviate inflammation by downregulating nuclear factor-kappa B (NFκB) and TNF-α, thereby reducing the risk of NAFLD^62^. Preformed vitamin A (such as retinol and retinyl esters) was not inversely associated with NAFLD was that with the increase of dietary retinol, the content of animal fat and cholesterol also increased, which was the risk factor for NAFLD. The exact mechanism for the association between total dietary vitamin A intake and NAFLD risk is still unclear. Additional studies were warranted to explore the underlying mechanisms.

Interestingly, some studies indicated that non-vitamin A intakes also have certain effect on NAFLD. A study showed that the increased incidence of NAFLD patients was closely related to the increased intake of cholesterol and saturated fat^63^. However, a Chinese study found a negative relationship between dietary vitamin C intake and NAFLD^64^. Another study revealed that dietary vitamin E intake was related to lower odds of NAFLD^65^. The study by Christensen reported that intake of α-carotene, β-carotene, β-cryptoxanthin, and lutein/zeaxanthin had a significant improvement in NAFLD^57^. In addition, Kaiyue et al.^66^ also reported that flavonoids can have beneficial effects on NAFLD by regulating the activity of CYP2E1.

The strength of our study is as follows. First, this study was a nationwide study conducted on American adults, which increased the statistical power and reliability of the study. Second, to explore the association between dietary vitamin A intake and NAFLD risk, the subgroup analyses stratified by gender and age were used. And provitamin A carotenoids intake was inversely associated with NAFLD, especially in women and those aged < 45 years among adult American in our study. Third, dose–response relationships between total dietary vitamin A intake and NAFLD risk were assessed using a restricted cubic spline regression.

Nevertheless, several limitations should be addressed. First, we did not determine the causal relationship between total dietary vitamin A intake and NAFLD risk because of the cross-sectional design of this study. Second, using data from two 24-h dietary recall interviews might lead to recall bias. In this study, dietary vitamin A intake from supplements was not included in total dietary vitamin A intake, so we did not evaluate the relationship between dietary vitamin A intake from supplements and NAFLD risk. Third, USFLI had superior sensitivity in identifying NAFLD patients^9,41^. However, USFLI cannot determine NAFLD stages, and the correlation between total dietary vitamin A intake and NAFLD risk is still unclear. Fourth, the possibility of residual confusion caused by other confounders cannot be excluded. Finally, NAFLD status was estimated based on a previously validated index rather than a clinical diagnosis.

## Conclusions

Under the general trend of the increasing global prevalence rate of NAFLD, US has become one of the high-prevalence-rate countries. To reduce the prevalence of NAFLD, targeted measures have been implemented. Although therapeutic options for NAFLD are limited, the current findings shed light on the prevention and treatment of NAFLD by identifying modifiable lifestyle factors, especially the consumption of provitamin A carotenoids.

### Supplementary Information

Supplementary Tables.

## Data Availability

The dataset generated for this study is available in online repositories, and access is provided through the following link: https://www.cdc.gov/nchs/nhanes/index.htm.
