# Supplementary material for: Association between dietary vitamin A intake from different sources and non-alcoholic fatty liver disease among adults
Source: Sci Rep. 2024 Jan 22;14:1851. doi: 10.1038/s41598-024-52077-5 (PMC10803811; doi:10.1038/s41598-024-52077-5)
Supplement: Supplementary file 1 — Supplementary Tables. [file 41598_2024_52077_MOESM1_ESM.docx]

**Supplementary Table1** Baseline characteristics of the participants by NAFLD, U.S. adults aged ≥20years, NHANES 2007-2014, stratified by gender.

| Group | NAFLD (male) | | p-value | NAFLD (female) | | p-value |
| --- | --- | --- | --- | --- | --- | --- |
|  | no | yes |  | no | yes |  |
| Age Group (n, %) |  |  | <0.001 |  |  | <0.001 |
| 20–44 years | 805(45.20%) | 388(30.17%) |  | 1085(45.06%) | 321(28.21%) |  |
| 45–59 years | 427(23.98%) | 328(25.51%) |  | 561(23.30%) | 327(28.73%) |  |
| 60–74 years | 346(19.43%) | 389(30.25%) |  | 516(21.43%) | 353(31.02%) |  |
| ≥75 years | 203(11.40%) | 181(14.07%) |  | 246(10.22%) | 137(12.04%) |  |
| Race/Ethnicity (n, %) |  |  | <0.001 |  |  | <0.001 |
| Mexican American | 204(11.45%) | 281(21.85%) |  | 284(11.79%) | 271(23.81%) |  |
| Other Hispanic | 175(9.83%) | 144(11.20%) |  | 269(11.17%) | 150(13.18%) |  |
| Non-Hispanic White | 789(44.30%) | 650(50.54%) |  | 1056(43.85%) | 505(44.38%) |  |
| Non-Hispanic Black | 394(22.12%) | 120(9.33%) |  | 537(22.30%) | 149(13.09%) |  |
| Other/Multiracial | 219(12.30%) | 91(7.08%) |  | 262(10.88%) | 63(5.54%) |  |
| BMI (n, %) |  |  | <0.001 |  |  | <0.001 |
| <25 kg/m^2^ | 736(41.44%) | 67(5.22%) |  | 980(40.71%) | 41(3.61%) |  |
| 25 to <30 kg/m^2^ | 779(43.86%) | 412(32.09%) |  | 789(32.78%) | 225(19.81%) |  |
| ≥30 kg/m^2^ | 261(14.70%) | 805(62.69%) |  | 638(26.51%) | 870(76.58%) |  |
| Educational Level (n, %) |  |  | 0.005 |  |  | <0.001 |
| < High school | 398(22.36%) | 396(30.84%) |  | 504(20.96%) | 406(35.74%) |  |
| High school | 418(23.48%) | 298(23.21%) |  | 536(22.29%) | 245(21.57%) |  |
| > High school | 964(54.16%) | 590(45.95%) |  | 1365(56.76%) | 485(42.69%) |  |
| Annual household income (n, %) |  |  | <0.001 |  |  | <0.001 |
| <$20,000 | 266(15.74%) | 235(19.03%) |  | 514(22.15%) | 326(29.94%) |  |
| $20,000–$44,999 | 549(32.49%) | 486(39.35%) |  | 803(34.60%) | 445(40.86%) |  |
| $45,000–$74,999 | 340(20.12%) | 237(19.19%) |  | 441(19.00%) | 172(15.79%) |  |
| ≥$75,000 | 535(31.66%) | 277(22.43%) |  | 563(24.26%) | 146(13.41%) |  |
| Smoking status (n, %) |  |  | <0.001 |  |  | <0.001 |
| Yes | 840(47.19%) | 705(54.82%) |  | 736(30.58%) | 426(37.43%) |  |
| No | 940(52.81%) | 581(45.18%) |  | 1671(69.42%) | 712(62.57%) |  |
| Vigorous recreational activity (n, %) |  |  | <0.001 |  |  | <0.001 |
| Yes | 571(32.06%) | 192(14.93%) |  | 460(19.10%) | 78(6.85%) |  |
| No | 1210(67.94%) | 1094(85.07%) |  | 1948(80.90%) | 1060(93.15%) |  |
| Hypertension (n, %) |  |  | <0.001 |  |  | <0.001 |
| Yes | 743(41.72%) | 812(63.14%) |  | 905(37.58%) | 727(63.88%) |  |
| No | 1038(58.28%) | 474(36.86%) |  | 1503(62.42%) | 411(36.12%) |  |
| Diabetes (n, %) |  |  | <0.001 |  |  | <0.001 |
| Yes | 229(12.86%) | 461(35.85%) |  | 276(11.46%) | 449(39.46%) |  |
| No | 1552(87.14%) | 825(64.15%) |  | 2132(88.54%) | 689(60.54%) |  |
| Cholesterol (mg/dL) | 186.24±38.60 | 191.36±42.80 | 0.001 | 196.55±42.49 | 197.47±40.39 | 0.539 |
| Uric Acid (mg/dL) | 5.79±1.18 | 6.45±1.34 | <0.001 | 4.66±1.17 | 5.57±1.38 | <0.001 |
| Average energy intake (kcal/day) | 2183.25±721.44 | 2132.44±732.41 | 0.056 | 1696.20±597.30 | 1662.96±596.28 | 0.122 |
| Total dietary vitamin A intake (RAEs, μg/1000kcal/day) | 307.46±319.64 | 301.54±247.22 | 0.579 | 361.73±252.23 | 352.98±310.36 | 0.372 |
| Preformed vitamin A intake (RAEs, μg/1000kcal/day) | 120.73±175.31 | 125.67±168.77 | 0.434 | 124.15±129.38 | 145.48±240.34 | 0.001 |
| Provitamin A carotenoids intake (RAEs, μg/1000kcal/day) | 171.02±264.89 | 158.06±174.73 | 0.126 | 219.35±215.27 | 186.14±187.02 | <0.001 |

| **Supplementary Table2** Weighted ORs and 95% CIs for NAFLD according to the quartiles of dietary retinol intake (μg/1000kcal/day), stratified by gender | | | |
| --- | --- | --- | --- |
|  | Crude | Model1 | Model2 |
|  | OR(95%CI) | OR(95%CI) | OR(95%CI) |
| Male |  |  |  |
| Total dietary vitamin A intake (RAEs, μg/1000kcal/day) |  |  |  |
| <172.88 | 1.00(ref.) | 1.00(ref.) | 1.00(ref.) |
| 172.88 to <257.52 | 1.16(0.86-1.55) | 1.06(0.78-1.44) | 1.14(0.79-1.65) |
| 257.52 to <379.31 | 1.02(0.76-1.36) | 0.86(0.63-1.18) | 0.97(0.67-1.41) |
| ≥379.31 | 1.03(0.80-1.33) | 0.84(0.63-1.13) | 0.98(0.68-1.41) |
| Preformed vitamin A intake (RAEs, μg/1000kcal/day) |  |  |  |
| <57.27 | 1.00(ref.) | 1.00(ref.) | 1.00(ref.) |
| 57.27 to <101.17 | 0.98(0.72-1.34) | 0.89(0.64-1.23) | 1.07(0.71-1.62) |
| 101.17 to <157.63 | 0.97(0.77-1.22) | 0.89(0.70-1.12) | 0.94(0.70-1.28) |
| ≥157.63 | 0.99(0.72-1.35) | 0.88(0.63-1.23) | 0.89(0.55-1.44) |
| Provitamin A carotenoids intake (RAEs, μg/1000kcal/day) |  |  |  |
| <60.97 | 1.00(ref.) | 1.00(ref.) | 1.00(ref.) |
| 60.97 to <118.88 | 1.00(0.77-1.31) | 0.99(0.75-1.30) | 1.08(0.76-1.54) |
| 118.88 to <219.33 | 0.87(0.71-1.07) | 0.81(0.65-1.01) | 0.97(0.68-1.40) |
| ≥219.33 | 0.90(0.65-1.23) | 0.81(0.57-1.14) | 1.04(0.74-1.48) |
| Female |  |  |  |
| Total dietary vitamin A intake (RAEs, μg/1000kcal/day) |  |  |  |
| <208.86 | 1.00(ref.) | 1.00(ref.) | 1.00(ref.) |
| 208.86 to <310.87 | 1.29(1.00-1.66) ^*^ | 1.18(0.92-1.52) | 1.18(0.84-1.65) |
| 310.87 to <455.24 | 0.95(0.76-1.20) | 0.84(0.66-1.06) | 0.74(0.56-0.99) ^*^ |
| ≥455.24 | 0.78(0.61-1.00) | 0.64(0.49-0.84) ^**^ | 0.84(0.58-1.21) |
| Preformed vitamin A intake (RAEs, μg/1000kcal/day) |  |  |  |
| <61.19 | 1.00(ref.) | 1.00(ref.) | 1.00(ref.) |
| 61.19 to <108.69 | 1.17(0.95-1.44) | 1.11(0.90-1.36) | 1.10(0.82-1.49) |
| 108.69 to <171.03 | 1.47(1.12-1.95) ^**^ | 1.34(1.01-1.77) ^*^ | 1.29(0.90-1.85) |
| ≥171.03 | 1.32(1.06-1.64) ^*^ | 1.18(0.94-1.48) | 1.06(0.80-1.41) |
| Provitamin A carotenoids intake (RAEs, μg/1000kcal/day) |  |  |  |
| <79.56 | 1.00(ref.) | 1.00(ref.) | 1.00(ref.) |
| 79.56 to <155.56 | 1.01(0.78-1.31) | 0.95(0.73-1.25) | 1.01(0.72-1.44) |
| 155.56 to <280.76 | 0.79(0.63-0.99) ^*^ | 0.72(0.56-0.92) ^*^ | 0.78(0.53-1.15) |
| ≥280.76 | 0.54(0.41-0.72) ^**^ | 0.46(0.34-0.62) ^**^ | 0.61(0.43-0.89) ^*^ |
| OR, odds ratio; CI, confidence interval. Model 1 adjusted for age. Model 2 adjusted for age, race, education level, smoking status, hypertension, diabetes, physical activity, income level, BMI, UA and TC. The lowest quartile of dietary retinol intake was used as the reference group. Results are survey-weighted. *^*^p<0.05, ^**^p<0.0*1. | | | |

| **Supplementary Table3** Weighted ORs and 95% CIs for NAFLD according to the quartiles of dietary retinol intake (μg/1000kcal/day), stratified by age | | | |
| --- | --- | --- | --- |
|  | Crude | Model1 | Model2 |
|  | OR(95%CI) | OR(95%CI) | OR(95%CI) |
| <45 years |  |  |  |
| Total dietary vitamin A intake (RAEs, μg/1000kcal/day) |  |  |  |
| <193.42 | 1.00(ref.) | 1.00(ref.) | 1.00(ref.) |
| 193.42 to <287.06 | 1.13(0.87-1.45) | 1.12(0.87-1.46) | 1.26(0.86-1.83) |
| 287.06 to <421.28 | 0.99(0.78-1.25) | 1.04(0.82-1.32) | 1.06(0.73-1.54) |
| ≥421.28 | 0.79(0.62-1.01) | 0.84(0.66-1.08) | 0.81(0.58-1.13) |
| Preformed vitamin A intake (RAEs, μg/1000kcal/day) |  |  |  |
| <60.11 | 1.00(ref.) | 1.00(ref.) | 1.00(ref.) |
| 60.11 to <105.52 | 1.07(0.82-1.40) | 1.09(0.83-1.43) | 1.05(0.68-1.61) |
| 105.52 to <167.31 | 1.45(1.17-1.79) ^**^ | 1.46(1.18-1.82) ^**^ | 1.36(0.99-1.88) |
| ≥167.31 | 1.22(0.91-1.63) | 1.25(0.94-1.68) | 1.13(0.75-1.68) |
| Provitamin A carotenoids intake (RAEs, μg/1000kcal/day) |  |  |  |
| <70.65 | 1.00(ref.) | 1.00(ref.) | 1.00(ref.) |
| 70.65 to <140.59 | 0.94(0.71-1.24) | 0.97(0.73-1.29) | 1.01(0.66-1.56) |
| 140.59 to <248.15 | 0.71(0.55-0.90) ^**^ | 0.73(0.57-0.94) ^*^ | 0.76(0.50-1.14) |
| ≥248.15 | 0.58(0.43-0.79) ^**^ | 0.62(0.46-0.84) ^**^ | 0.60(0.42-0.87) ^**^ |
| ≥45 years |  |  |  |
| Total dietary vitamin A intake (RAEs, μg/1000kcal/day) |  |  |  |
| <189.62 | 1.00(ref.) | 1.00(ref.) | 1.00(ref.) |
| 189.62 to <282.96 | 1.13(0.90-1.41) | 1.16(0.92-1.46) | 1.07(0.74-1.55) |
| 282.96 to <417.88 | 1.15(0.94-1.40) | 1.21(0.99-1.48) | 1.06(0.81-1.40) |
| ≥417.88 | 0.78(0.63-0.97) ^*^ | 0.85(0.69-1.06) | 0.98(0.74-1.29) |
| Preformed vitamin A intake (RAEs, μg/1000kcal/day) |  |  |  |
| <58.49 | 1.00(ref.) | 1.00(ref.) | 1.00(ref.) |
| 58.49 to <104.55 | 1.13(0.87-1.47) | 1.13(0.88-1.45) | 1.20(0.85-1.71) |
| 104.55 to <162.63 | 1.08(0.85-1.38) | 1.10(0.86-1.40) | 0.99(0.73-1.35) |
| ≥162.63 | 1.05(0.83-1.34) | 1.08(0.85-1.38) | 0.95(0.67-1.35) |
| Provitamin A carotenoids intake (RAEs, μg/1000kcal/day) |  |  |  |
| <70.14 | 1.00(ref.) | 1.00(ref.) | 1.00(ref.) |
| 70.14 to <136.52 | 0.96(0.73-1.26) | 0.97(0.73-1.27) | 1.15(0.83-1.60) |
| 136.52 to <257.54 | 0.98(0.73-1.30) | 1.01(0.75-1.35) | 1.12(0.79-1.57) |
| ≥257.54 | 0.68(0.53-0.88) ^**^ | 0.73(0.57-0.94) ^*^ | 1.01(0.75-1.36) |
| OR, odds ratio; CI, confidence interval. Model 1 adjusted for gender. Model 2 adjusted for gender, race, education level, smoking status, hypertension, diabetes, physical activity, income level, BMI, UA and TC. The lowest quartile of dietary retinol intake was used as the reference group. Results are survey-weighted. *^*^p<0.05, ^**^p<0.0*1. | | | |
